# Acoustic Angiography: A New Imaging Modality for Assessing Microvasculature Architecture

**DOI:** 10.1155/2013/936593

**Published:** 2013-07-17

**Authors:** Ryan C. Gessner, C. Brandon Frederick, F. Stuart Foster, Paul A. Dayton

**Affiliations:** ^1^UNC and NCSU Joint Department of Biomedical Engineering, 304 Taylor Hall, 109 Mason Farm Road, Chapel Hill, NC 27599-6136, USA; ^2^Department of Medical Biophysics, University of Toronto, Sunnybrook Health Sciences Centre, 2075 Bayview Avenue, Toronto, ON, Canada M4N 3M5

## Abstract

The purpose of this paper is to provide the biomedical imaging community with details of a new high resolution contrast imaging approach referred to as “acoustic angiography.” Through the use of dual-frequency ultrasound transducer technology, images acquired with this approach possess both high resolution and a high contrast-to-tissue ratio, which enables the visualization of microvascular architecture without significant contribution from background tissues. Additionally, volumetric vessel-tissue integration can be visualized by using b-mode overlays acquired with the same probe. We present a brief technical overview of how the images are acquired, followed by several examples of images of both healthy and diseased tissue volumes. 3D images from alternate modalities often used in preclinical imaging, contrast-enhanced micro-CT and photoacoustics, are also included to provide a perspective on how acoustic angiography has qualitatively similar capabilities to these other techniques. These preliminary images provide visually compelling evidence to suggest that acoustic angiography may serve as a powerful new tool in preclinical and future clinical imaging.

## 1. Introduction

Blood vessel structure and patency are known to be related to the state and progression of many diseases [1, 2]. Abnormal vessel and vessel network morphologies have been positively correlated to malignancy across species [3, 4]. In preclinical studies of the disease and drug research, there are several noninvasive imaging modalities that can be utilized to visualize blood vessel structure. These are magnetic resonance imaging (MRI), computed tomography (CT), ultrasound, and more recently—photoacoustic imaging. To select one of these imaging methods, researchers must compromise between the variables of system including study cost, data acquisition time, ionizing radiation dose, imaging depth, and image resolution. High-field MRI is the most expensive, requires the longest acquisition times, and requires a dedicated facility for shielding and maintenance. CT is also fairly expensive, bulky, and primarily limited by a high ionizing radiation dose. Ultrasound is the least expensive, the most portable, and provides the fastest image acquisition. Photoacoustic imaging has similar portability to ultrasound and can provide high resolution images of the microvasculature as well as additional functional information (such as blood oxygen saturation), but is the most limited in penetration depth among these modalities. 

 In the past, the reputation of ultrasound has been as a modality with limited ability to assess microvasculature structure. However, recent advances in high frequency ultrasound technology have substantially improved image quality to the point that it has become a favored technique for many researchers studying small animal models. While conventional high frequency ultrasound does still lack the resolution and contrast of recently published CT [5] and MR studies [6], it has been effectively implemented to monitor disease progression and therapeutic responses in many different cancer models, including pancreatic [7], prostate [8], melanoma [9], ovarian [10], lung [11], and mammary [12] cancer models. 

 Traditional noncontrast-enhanced ultrasound excels at imaging anatomical features, but is challenged when imaging blood flow in small vessels due to weak ultrasound scatter from blood components. Thus, for imaging blood flow in small vessels with high sensitivity, clinical ultrasound relies on the application of intravascular microbubble contrast agents (MCAs). Because of the nonlinear behavior of MCAs in response to an ultrasound pulse, their acoustic scatter can be detected and separated from tissue enabling image and signal processing techniques to segment blood vessels from the tissue background. Most nonlinear imaging techniques are most efficient near the resonant frequencies of microbubble contrast agents, typically less than 10 MHz, which is a good match for clinical ultrasound imaging applications. However, for the improved imaging resolution required in small-animal imaging, high frequency ultrasound systems typically utilize frequencies in the 20–60 MHz range, substantially above the resonant frequencies for most microbubbles. Typically, high frequency imaging systems have been challenged to perform nonlinear contrast imaging for this reason.

 Here, we report the technique of high resolution microvascular imaging using a prototype dual-frequency transducer which enables vascular imaging with both high contrast sensitivity and imaging resolution. With this technology, the above-mentioned resolution versus contrast-sensitivity tradeoff can be partially circumvented by detecting the high frequency components from the wideband acoustic energy produced by MCAs when they are excited at low frequencies near their resonance. The potential of this technique was initially demonstrated by Kruse and Ferrara [13], where it was observed that microbubbles excited with short pulses at 2.25 MHz produced broadband energy that exceeded 45 MHz. The main challenge of this approach to date, however, has been the lack of available transducers that could transmit energy at low frequencies (such as 2–4 MHz) to excite microbubbles and, at the same time confocally detect scattered echoes in the 15–45 MHz bandwidth. 

 Our prototype transducer consists of both low and high frequency confocal elements, in contrast to standard ultrasound imaging transducers which typically operate within a single frequency range. The low frequency element (4 MHz) excites MCAs near their resonance, and the high frequency element (30 MHz) receives high frequency content from the excited microbubbles. The broadly separated frequency bandwidths of the two elements used in our system are what differentiate this approach from the dual-frequency probes previously presented by other groups [14–16] and are what enable its high resolution and high contrast imaging ability. This approach enables detection of signal from microbubble contrast with almost complete tissue suppression. Furthermore, because of the high frequency receive, the system is able to achieve high resolution, yet with less attenuation than systems operating with high frequency on both transmit and receive. The resulting ultrasound images illustrate high resolution depictions of the microvasculature, without background from surrounding tissue, not unlike X-ray angiography images. Furthermore, through use of traditional single-frequency pulse-echo, tissue b-mode images can be acquired for anatomical reference. Thus, we refer to images acquired with this technology as “acoustic angiography”. In this paper we discuss the prototype transducer and present example of high resolution in vivo 3D images, discussing how these images could be used for diagnostics. For qualitative comparison, we also provide contrast-enhanced CT and photoacoustic images to illustrate that acoustic angiography could be considered as an alternative to these other techniques.

## 2. Materials and Methods

### 2.1. Prototype Imaging System

**Figure 1:**
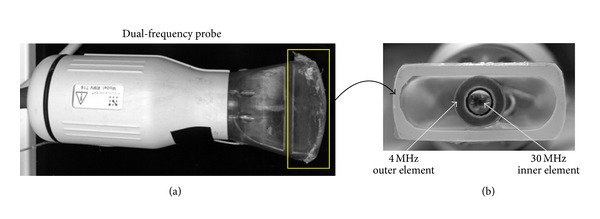
Photograph of the dual-frequency probe, illustrating the side view (a) and the bottom view (b) showing the two transducer elements. The probe housing is a modified VisualSonics “RMV” transducer housing.

 The custom dual-frequency transducer was fabricated by integrating a high frequency broadband 30 MHz transducer element (RMV 707, VisualSonics, Toronto, ON, Canada) with a concentric low frequency 4 MHz annulus (Figure 1), as previously described [17]. The radius of curvature for the 4 MHz element was 12.7 mm, which matched that of the 30 MHz receiver element for overlapping depth of field. The selection of the 30 MHz element was made to ensure that both rats and mice could be imaged with good spatial resolution, while the 4 MHz pulsing frequency was selected because it is near the resonance frequency of many of the contrast agents utilized by our lab. It is likely that other frequency ranges would be optimal for other contrast agents, or applications that require different depths of penetration. The electrical return path from both elements was isolated so that the transmitter could be driven by an external pulser. To drive the system, we utilized a commonly implemented high frequency preclinical Vevo770 ultrasound scanner (VisualSonics, Toronto, ON, Canada) that had been modified to work with our custom dual-frequency probe. The Vevo770 was altered by insertion of a 10 MHz high-pass filter (TTE, Los Angeles, CA, USA) after the receiver amplifier but before analog to digital conversion. This served to ensure that no tissue signal leaked into the data during dual-frequency imaging. The trigger was synchronized to an external arbitrary waveform generator (AWG 2021, Tektronix, Beaverton, OR, USA) to drive the low frequency element. Signals from the waveform generator were amplified by 55 dB through a RF amplifier (ENI, Rochester, NY, USA) before exciting the transmitter. 

### 2.2. Characterization of the Probe

**Figure 2:**
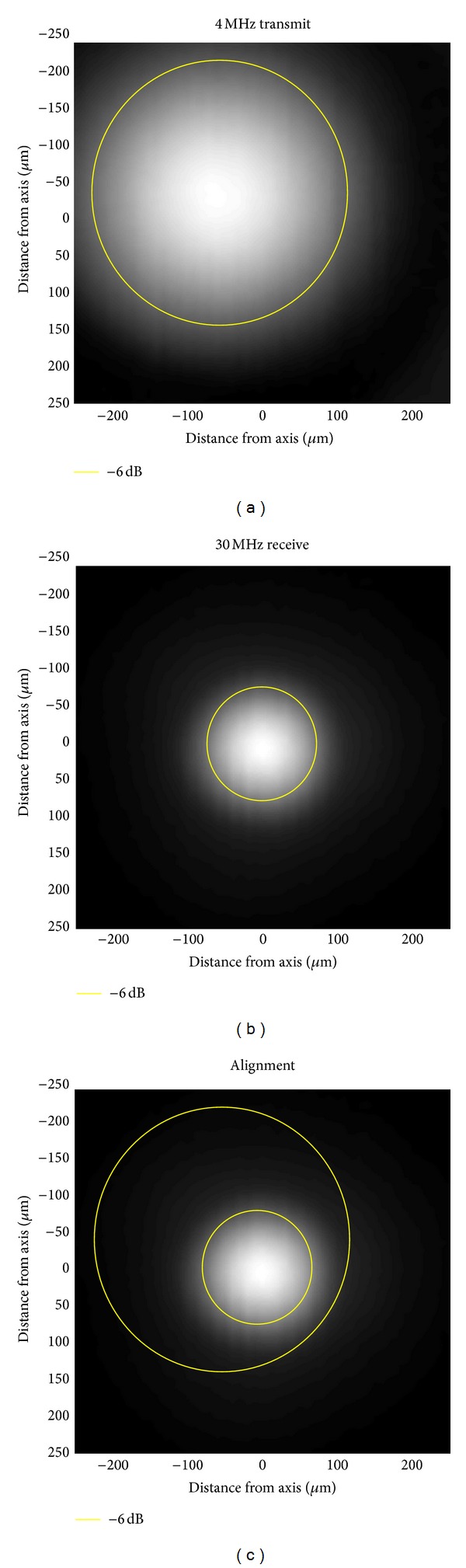
Beam plots showing the −6 dB beamwidth for the (a) 4 MHz (298 *μ*m) and (b) 30 MHz (137 *μ*m) elements. Panel (c) illustrates the confocal alignment of the transducers, where the circles approximate the −6 dB regions of the two transducers. There was a misalignment of approximately 90 *μ*m of the two transducers on this prototype probe due to the challenge of the manufacturing process. Improving the element focal alignment is an obvious way to improve the performance of this system in future iterations.

 Beam-field mapping with a needle hydrophone enabled determination of the −6 dB beamwidths of the transmit and receive transducer (Figure 2). The −6 dB lateral beamwidth for the 4 MHz element was 298 *μ*m, and the −6 dB lateral beamwidth for the 30 MHz element was 137 *μ*m. During this procedure, it was observed that the two elements were not exactly confocal, and the lateral focal zones were actually misaligned by 90 *μ*m. Thus, the −6 dB focal spot of the 30 MHz element was centered along the edge of the −6 dB focal spot of the 4 MHz transducer (Figure 2(c)). This misalignment was due to the challenge of the manufacturing process and will be an area for improvement in future prototypes. The axial −6 dB beamwidths of the two transducers were 7.05 mm and 1.99 mm, for 4 MHz and 30 MHz, respectively. The focal spot misalignment in the axial direction was only 0.9 mm. It is anticipated that with correction to these misalignments, future probes will be able to provide similar or improved sensitivity at reduced excitation energies.

 Transmission with the high frequency (30 MHz) element allowed traditional high frequency b-mode imaging in order to obtain anatomical images, whereas transmission with the low frequency (4 MHz) element enabled Acoustic Angiography to obtain contrast only images. 

**Figure 3:**
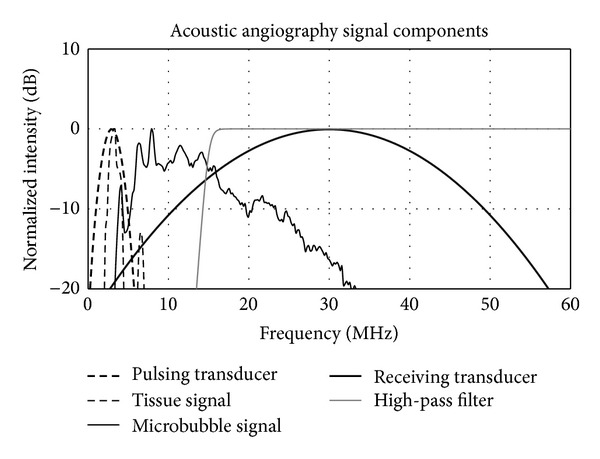
A frequency domain representation of the signals of all transmit and receive components of acoustic angiography imaging. The tissue and bubble responses were acquired using an experimental system described previously [17] and are displayed here to illustrate the relationship between the bandwidths and the two transducer elements as well as the tissue and bubble responses, for example, acoustic angiography imaging conditions.

 Both the 4 MHz and 30 MHz transducers had approximately 100% bandwidth. A frequency domain representation approximating the probe's two transducer bandwidths, the filter cutoff, and example tissue and microbubble responses can be found in Figure 3. 

 Images were acquired and saved on the ultrasound system, then later exported for offline analysis. All images were acquired in 3D image using a linear translational motor stage synchronized with the frame trigger. 

### 2.3. Animal Imaging

 All animal studies were performed in rats with a protocol approved by the University of North Carolina at Chapel Hill Institutional Animal Care and Use Committee. Seven animals were imaged for this study: three animals with a flank tumor model, three healthy controls imaged in the same region, and one animal imaged for a healthy kidney for comparison to contrast-enhanced CT imaging. The tumor model (syngeneic fibrosarcoma: “FSA”; [18]) was initiated by propagating tumor tissue through subcutaneous implant in the rodent flank. Initial tumor samples were graciously provided by the Dewhirst Lab at Duke University. The acoustic angiography strategy necessitates the use of contrast agents to provide signal from the vasculature. In these studies, microbubble contrast agent with a polydisperse diameter distribution centered at 0.9 *μ*m with a standard deviation of 0.45 *μ*m was prepared as previously described [19]. Microbubble contrast was diluted in saline and administered via a tail vein catheter with a syringe pump at 70 *μ*L/min at a concentration of 3.3*·*10^9^ bubbles/mL. The administration of contrast began 20 seconds prior to imaging; this waiting period would be sufficient to for the initial bolus of contrast to perfuse the tissue prior to image acquisition. Imaging pulses were 4 MHz single cycle sinusoids with a peak negative pressure of 1.23 MPa (mechanical index = 0.62). 3D ultrasound images were acquired with interplane step sizes of 150–200 *μ*m and required fewer than 3 minutes to acquire. Since the tissues of interest were in the lower abdomen and flank, respiratory motion artifacts were limited. Acquisition of all ultrasound images was coplanar with the axial anatomical plane while the animals were in dorsal recumbency. 

 The CT images were acquired of the rat kidney 45 minutes after the injection of contrast. The contrast agent implemented was Fenestra VC (Advanced Research Technologies, Montreal, QC, Canada), an iodinated lipid blood pool CT contrast agent injected at a concentration of 3 mL/450 g tissue. CT images were acquired using the GE eXplore SpeCZT CT 120 SPECT/CT (GE Healthcare, London, ON, Canada) at 90 kVp using 900 views over 360 degrees with total output 576 mAs. The total dose delivered was 300 mGy. Reconstruction was performed on 100 *μ*m isotropic grid with a standard Feldkamp-Davis-Kress algorithm. 

 The photoacoustic images were reproduced with permission from work by Zhang et al. [20]. That study implemented a photoacoustics system using an excitation wavelength of 590 nm, a 38 *μ*m thick polymer ultrasound transducer with a −3 dB acoustic bandwidth of 22 MHz, and a frequency response characterized by a smooth roll-off with its zero response occurring at 58 MHz [20].

## 3. Results

**Figure 4:**
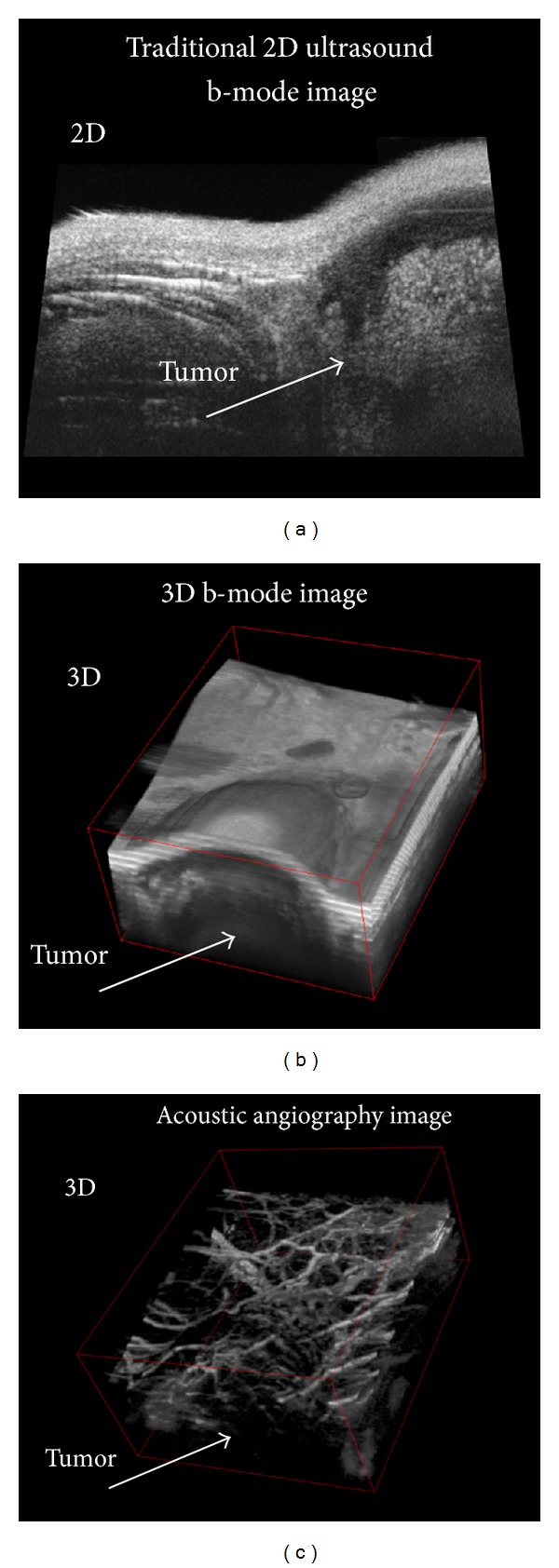
(a) 2D and (b) 3D “b-mode” ultrasound images from the same sample volume illustrating a subcutaneous tumor in a rat. (c) “acoustic angiography” image of the same sample volume as (b) acquired with dual-frequency transducer, illustrating contrast-only image, depicting microvasculature.

Imaging studies with our dual-frequency transducer immediately demonstrated a new paradigm in ultrasound imaging. Images illustrated signal from contrast only, with virtually no tissue signal (Figure 4). The result was images of tissue microvascular structure; hence, we refer to this technique as “acoustic angiography.” 

**Figure 5:**
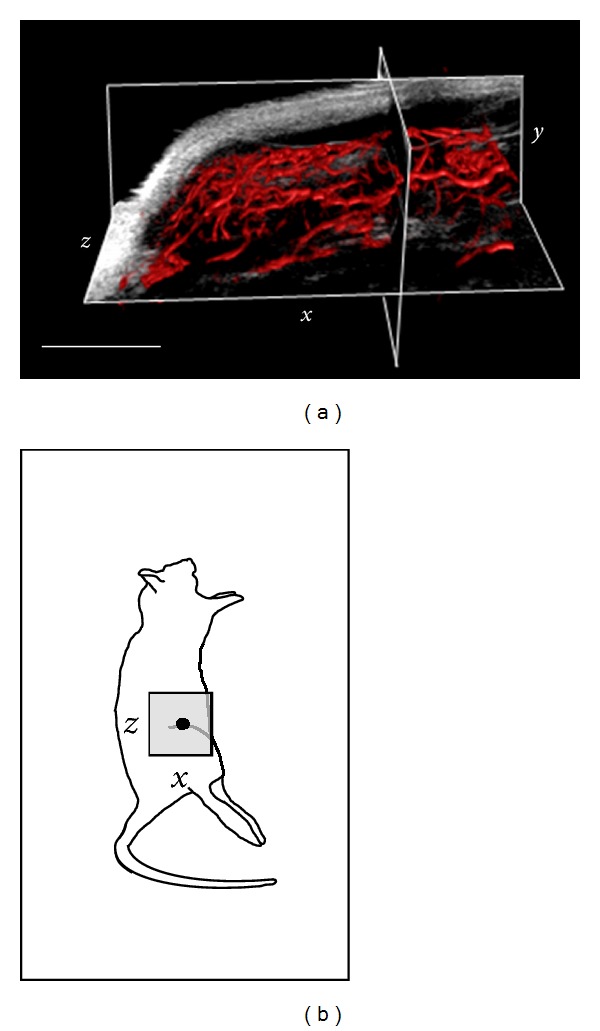
(a) An overlay of the microvasculature within a tumor provided by acoustic angiography (red) onto a tissue-only image provided by high frequency b-mode (grayscale). This figure was created using 3D slicer (National Institutes of Health) with a 3D rendering of the acoustic angiography data simultaneously displayed with a grayscale orthoslice of the b-mode data. Displaying data in this fashion illustrates microvessel and tissue morphologies, as well as vascular-tissue integration. Scale bar = 0.5 cm. (b) A cartoon illustrates the approximate location of this 3D image volume.

 One likely application of acoustic angiography is visualization of tumor microvascular structure with anatomical reference.  Figure 5(a) illustrates the ability of the probe to acquire both vascular images and tissue images of the same in vivo sample volume. Vessels imaged in acoustic angiography mode are overlaid with the traditional high-frequency b-mode image, thereby enabling the visualization and spatial characterization of vessel-tissue integration. 

**Figure 6:**
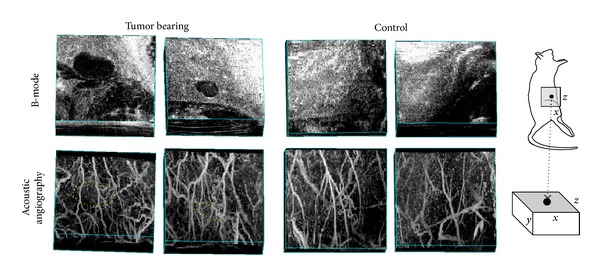
Multiple comparisons of 3D tissue volumes: two containing a tumor (left) and two healthy controls (right). The bottom images are acoustic angiography maximum intensity projections, while the top images are b-mode acquisitions of the same tissue volumes. The tortuous and chaotic morphologies of the vessels in the presence of a lesion are contrasted by the relatively homogeneous vasculature in the healthy volume (tumor boundaries delineated in acoustic angiography image with dotted line). Image volumes = ~0.75 × 1.25 × 1.5 cm (axial, lateral, and elevational). A cartoon on the right indicates the imaging location and orientations of image volumes.

Figure 6 depicts both b-mode and acoustic angiography images of the microvasculature surrounding two different tumors versus microvasculature from two normal tissue volumes. Differences between the vascular architecture within these two types of tissue volumes are observable. Highly tortuous vessel structure is typical of angiogenic tumor vasculature [3, 4] and is not observed with as much frequency in the data for the healthy flank as it is in the tumor-bearing tissue volumes (Figure 6). Further analysis of microvasculature morphology is outside of the scope of this introductory paper, though these images provide compelling evidence of the possibility of applying this imaging technique toward quantitative approaches to assessing vessel architecture.

### 3.1. Acoustic Angiography in Comparison to Photoacoustic Imaging

**Figure 7:**
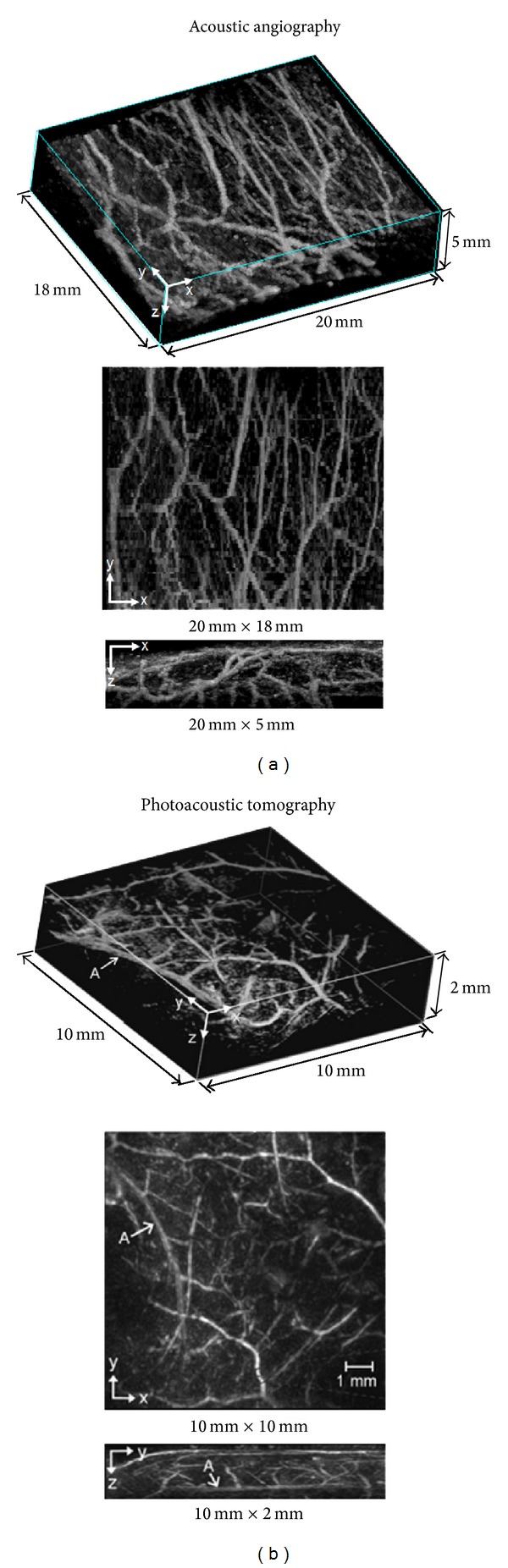
Comparison of acoustic angiography (a) to a photoacoustic imaging approach (b). The orientations of the acoustic angiography images have been purposefully arranged to mimic the photoacoustic images (adapted, with permission, from authors [20]) to allow a quick comparison between the two modalities. The imaging location of the acoustic angiography image was similar to that presented in Figure 6.

 Currently, photoacoustics is the gold standard noninvasive imaging technique for visualizing superficial microvascular structure. However, acoustic angiography has the potential to acquire similar images of microvessel networks. To qualitatively compare acoustic angiography images against images acquired with a photoacoustic imaging system, we present data of subcutaneous microvasculature in rodents with both imaging methods.  Figure 7(a) illustrates vasculature in the lower abdomen of a rat acquired with contrast ultrasound at transmit 4 MHz and at 30 MHz receive.  Figure 7(b) illustrates vasculature in the abdomen of a mouse obtained by photoacoustics as acquired by Zhang et al. [20]. In this image, the photoacoustic resolution was approximately 90 *μ*m (depth) by 130 *μ*m (lateral), and the imaging depth of field was 2 mm. The acoustic angiography images are of lower resolution (approximately 150 *μ*m (depth) by 200 *μ*m (lateral)); however, they illustrate a greater depth of penetration in this particular data set (5 mm in this case). Note that this comparison is not intended to suggest that one modality has superior resolution or penetration depth than the other; however, rather, our goal is to illustrate that acoustic angiography provides pulse-echo ultrasound with a method to achieve maps of vascular structure on a resolution and depth scale which were previously only available through photoacoustics. Although not presented here, newer photoacoustics techniques have illustrated sub-100 *μ*m spatial resolution at depths of almost 10 mm [21]. Similarly, we estimate that acoustic angiography could be achieved at depths up to several centimeters by reducing the receiving frequency, albeit at the expense of a reduced spatial resolution and with more background noise from tissue. Due to our transducer's fixed focus, we were unable to fully characterize limits on resolution at deeper depths, although this will be a focus of future work.

### 3.2. Acoustic Angiography in Comparison to Micro-CT

**Figure 8:**
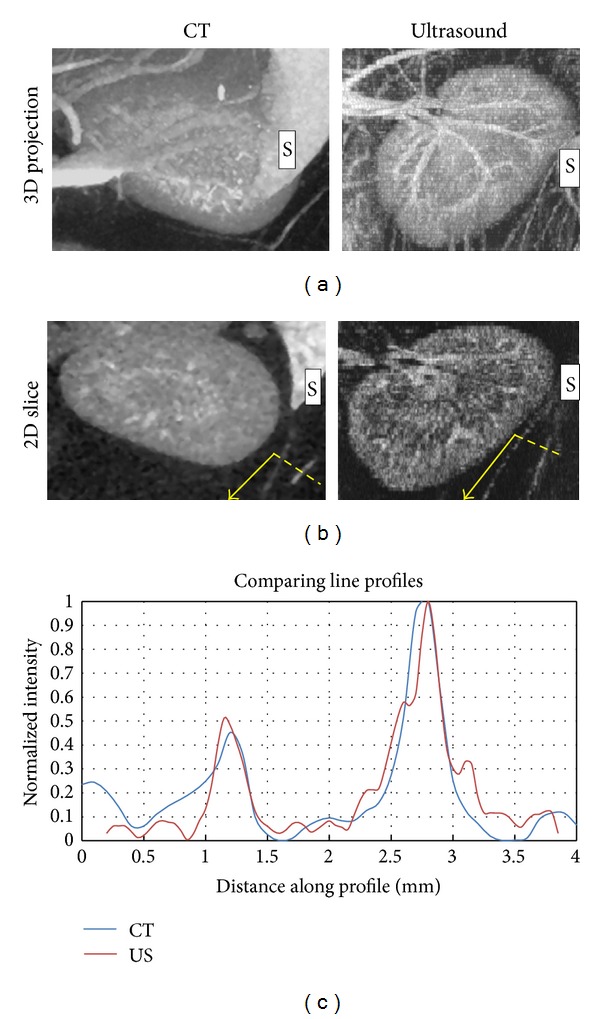
A comparison between the CT and ultrasound images acquired of the same kidney. The slight anatomical warping due to ultrasound probe contact with tissue resulted in a lack of direct one-to-one visual correspondence between the two 3D images, though the same anatomy is contained within each image. (a) Maximum intensity projections of image data. The spleen is visible in each of these images to the right of kidney (indicated with an “S”). (b) En face cuts through the 3D image. (c) Line profiles (dashed yellow lines) across two corresponding vessels near the spleen (dashed yellow lines) suggest similar feature sensitivity.

 We also qualitatively compared acoustic angiography to contrast-enhanced CT, which has also been used for microvascular imaging. Coronal maximum intensity projections (MIPs) through the ultrasound and CT acquisitions of the same kidney were performed (Figure 8). The CT dataset was reconstructed with a 100 *μ*m voxel grid, which resulted in an improved SNR, compared to the ultrasound image's 50 *μ*m voxel grid. Despite the slightly lower resolution of the ultrasound, previously measured to be approximately 150 *μ*m [17], the two imaging techniques illustrate similar anatomical microvascular features within the rat kidney. This is illustrated within the line profiles in Figure 8(c). These profiles were intensity normalized for display purposes and illustrate analogous spatial resolutions, albeit with slightly higher noise in the ultrasound signal. This increase in noise is expected, due to the 63% smaller voxel size in the ultrasound image (50 × 50 × 150 *μ*m compared to the CT's 100 × 100 × 100 *μ*m voxels).

## 4. Discussion and Summary

 The new contrast imaging technology demonstrated herein, which we refer to as acoustic angiography, has the capability to obtain images of microbubbles at high frequencies traditionally not utilized for nonlinear contrast imaging. The resulting images thus illustrate high resolution (~150 *μ*m) contrast-enhanced images of superficial microvasculature. Due to the tissue suppression achieved by detection of only harmonic energy well above the range produced by tissue, the resulting data also illustrates a high contrast-to-tissue ratio. 

 Our prototype transducer utilized a 4 MHz transmit, selected to take advantage of the resonant frequencies of the contrast agents utilized in our lab and 30 MHz receive for rodent imaging. It is likely that other transmit and receive combinations would be more optimal for other contrast agents, or for different depths of penetration. The tradeoff between resolution, depth of penetration, and signal separation are limitations of the acoustic angiography imaging approach. As with all high frequency ultrasound imaging methods, penetration depth is limited to a few centimeters due to the increased absorption of sound at high frequencies. One-way attenuation may be an advantage of this technique, as the high frequency components are only attenuated on receive since the bubble acts as the source. However, this is also a limitation as there is limit to how aggressively the microbubbles can be driven. In a prior study with detection of high-frequency content from microbubbles received with a similar 30 MHz transducer, it was observed that microbubble signal in response to 2 MHz excitation increased until an MI of approximately 0.45, after which it leveled out, likely when microbubble destruction began to dominate [17]. As the receiving frequency is reduced, penetration depth increases, yet our ability to separate signal from tissue and contrast is also reduced. For our prototype probe, an additional limitation was the fixed focus of the transducer's current form factor, which also provided a fairly narrow depth of field (~1 cm). Dual or multifrequency arrays with dynamic focusing will alleviate this problem in the future. 

 We suggest that acoustic angiography could perform similarly to micro-CT, MRI, or photoacoustic imaging in certain scenarios. Ultrasound imaging can be low cost and high throughput and avoids some of the drawbacks of CT and MR, such as ionizing radiation dose (CT), and high cost, and long acquisition times (MR). Both photoacoustic imaging and acoustic angiography have similar tradeoffs between resolution and depth of penetration. However, photoacoustic imaging has the additional advantages that it does not require a contrast agent, and that photoacoustic data can be used to derive other parameters about the target tissue, such as oxygen saturation. However, acoustic angiography may present an advantage of cost and simplicity over photoacoustics, since the latter requires a high power laser for signal generation. Furthermore, it might also be possible to combine acoustic angiography with targeted microbubbles for molecular imaging coregistered with microvascular features. This concept will require further evaluation to determine if acoustic angiography can be performed at low enough energies as to not destroy adherent targeted contrast agents. 

 The suppression of tissue signal and microvascular imaging ability demonstrated by the images within this paper provide motivation for the development of additional transducer designs to implement acoustic angiography. One obvious application of acoustic angiography is as a high throughput imaging technique for preclinical studies where there is a need for microvascular analysis, since the imaging resolution and depth are well suited to image rodents. 

Preclinical arrays could be designed for larger animal models, such as for rabbit or dog imaging. It is possible that in each of these clinical and preclinical applications the presence of disease-associated vascular abnormalities could be detected by acoustic angiography, as could the modifications to these vascular networks (such as the elimination or normalization of tumor vasculature) by effective therapeutic regimens. In the future, this technique may also find a role in clinical imaging of superficial tissues or tumors. 

Clinical implementation of this technique will depend on both the commercialization of appropriate hardware, as well as the increased regulatory acceptance of ultrasound contrast. While microbubbles are utilized for several oncology applications in Europe and Asia, they are still only approved for use by the United States Food and Drug Administration for cardiac applications. Nevertheless, data suggest that ultrasound contrast agents are very safe [22, 23], and it is likely that contrast ultrasound techniques will continue to gain increasing approval worldwide as they evolve to provide new information beyond that attainable with noncontrast techniques.
